# Identification of Pulse Onset on Cerebral Blood Flow Velocity Waveforms: A Comparative Study

**DOI:** 10.1155/2019/3252178

**Published:** 2019-07-02

**Authors:** Shadnaz Asgari, Nicolas Canac, Robert Hamilton, Fabien Scalzo

**Affiliations:** ^1^Department of Biomedical Engineering, California State University, Long Beach, CA 90840, USA; ^2^Department of Computer Engineering and Computer Science, California State University, Long Beach, CA 90840, USA; ^3^Neural Analytics, Inc., Los Angeles, CA 90064, USA; ^4^Department of Neurology, University of California, Los Angeles, CA 90095, USA; ^5^Department of Computer Science, University of California, Los Angeles, CA 90095, USA

## Abstract

The low cost, simple, noninvasive, and continuous measurement of cerebral blood flow velocity (CBFV) by transcranial Doppler is becoming a common clinical tool for the assessment of cerebral hemodynamics. CBFV monitoring can also help with noninvasive estimation of intracranial pressure and evaluation of mild traumatic brain injury. Reliable CBFV waveform analysis depends heavily on its accurate beat-to-beat delineation. However, CBFV is inherently contaminated with various types of noise/artifacts and has a wide range of possible pathological waveform morphologies. Thus, pulse onset detection is in general a challenging task for CBFV signal. In this paper, we conducted a comprehensive comparative analysis of three popular pulse onset detection methods using a large annotated dataset of 92,794 CBFV pulses—collected from 108 subarachnoid hemorrhage patients admitted to UCLA Medical Center. We compared these methods not only in terms of their accuracy and computational complexity, but also for their sensitivity to the selection of their parameters' values. The results of this comprehensive study revealed that using optimal values of the parameters obtained from sensitivity analysis, one method can achieve the highest accuracy for CBFV pulse onset detection with true positive rate (TPR) of 97.06% and positive predictivity value (PPV) of 96.48%, when error threshold is set to just less than 10 ms. We conclude that the high accuracy and low computational complexity of this method (average running time of 4ms/pulse) makes it a reliable algorithm for CBFV pulse onset detection.

## 1. Introduction

Cerebral hemodynamic impairment is prevalent in a wide range of neurological disorders, including subarachnoid hemorrhage, stroke, and traumatic brain injury [1]. In neurocritical care, accurate assessment of cerebral hemodynamics can help with effective planning of interventions and treatments to reduce secondary brain injury [2, 3]. The current gold standard for the measurement of cerebral blood flow (CBF) is positron emission tomography, a complicated and expensive modality which exposes patients to ionizing radiation [4]. Other neuroimaging techniques such as magnetic resonance perfusion are less complicated, but they cannot provide an assessment of cerebral hemodynamics in a continuous fashion [5].

Transcranial Doppler (TCD) ultrasound is a noninvasive, portable, and relatively inexpensive modality that allows for the continuous bedside monitoring of cerebral blood flow velocity (CBFV) as a surrogate measure of CBF. To obtain CBFV signal, a low-frequency (≤ 2 MHz) transducer probe is used to insonate the basal cerebral arteries through thin-bone trans-temporal windows [6]. Since its introduction in the early 80s, TCD studies have been widely used to evaluate cerebral hemodynamics in various conditions including impaired vasomotor function [7], sickle cell disease [8], subarachnoid hemorrhage [9], brain stem death [10], and intraoperative monitoring [11]. The analysis of CBFV pulse waveform morphology can help with the detection of vasospasm, hyperdynamic flow states, and increased cerebrovascular resistance [3, 6, 12, 13]. Furthermore, several recent studies have also shown that intracranial pressure (ICP) could be estimated noninvasively by relating the measurements of CBFV—and arterial blood pressure (ABP)—to the ICP [14–20].

The analysis of CBFV signal is challenging, in general. The cerebrovascular system is a nonlinear and nonstationary system. Thus, application of many conventional time-frequency analyses, developed based on the assumption of linearity and stationarity, may be less reliable [21]. Furthermore, given the difficulty of the insonation of the major cerebral arteries through the bone plate of the skull (especially for those with ticker temporal window), the quality of the collected CBFV is highly dependent on the TCD technician's competency and understanding of the three-dimensional cerebrovascular anatomy [6, 22]. Several other sources of noise and artifacts could also contaminate the CBFV signal, e.g., motion artifacts (mainly due to a commonly handheld TCD transducer) and cardiac and respiratory interferences [23]. These noises and artifacts can result in random baseline drift and perturbation of the CBFV pulse morphology. Thus, comparing to other physiological signals such as ICP and ABP, the CBFV signal has a relatively lower signal-to-noise ratio and less clear temporal pattern [24, 25].

Accurate beat-to-beat delineation of a pulsatile signal using its pulse onset (foot of the pulse) is an essential step for extraction and tracking of the pulse waveform morphology. Despite the existence of various pulse onset detection methods for pulsatile signals such as ABP and photoplethysmogram (PPG) signals [26–31], no method was exclusively tested on or adapted for the CBFV onset detection without additional information from other sources or signals such as electrocardiogram (ECG). Our group recently proposed a CBFV pulse onset detection method [32] based on adaptive thresholding, a state-of-the-art pulse onset detection originally developed for PPG signal [33]. We showed that our proposed method (Let us call it* Asgari method*) achieves a promising performance with true positive rate (TPR) and positive predictive value (PPV) of above 90%. To further enhance the performance of the CBFV pulse onset detection, in this work, we study the adaptation of two other state-of-the-art PPG onset detection methods (*Chen method *[27], and* Farooq method *[29]) for the CBFV signal using a large dataset of annotated CBFV pulses. To complete the study, we compare the performances of these three methods in terms of detection accuracy, computational complexity, and their sensitivity to the selection of parameters values. The result of this comprehensive analysis can enhance the automatic extraction and tracking of CBFV pulse morphology for noninvasive and continuous assessment of cerebral hemodynamics.

## 2. Materials and Methods

### 2.1. Patient Data

Our dataset consisted of more than 18 hours of CBFV and ECG data collected from 108 subarachnoid hemorrhage patients (66 males; age range 30-64, age average 48) that were admitted into Ronald Reagan UCLA Medical Center. The patients consented for allowing their data to be analyzed under the protocol as approved by the UCLA Internal Review Board. Simultaneous cardiovascular monitoring was performed using the bedside GE monitors and CBFV was measured using TCD machine (Multi-Dop X, Compumedics DWL, Singen, Germany). CBFV and ECG signals were recorded at a sampling rate of 400 Hz using a mobile cart at the bedside that was equipped with the PowerLab TM SP-16 data acquisition system (ADInstruments, Colorado Springs, CO). Note that ECG data in this study was solely used to guide the accurate annotation of CBFV pulse onsets. CBFV signal was segmented (into 92,794 pulses) using the ECG R-peak locations and then foot of each pulse was obtained as its minimum point. The average length of the rendered pulses was 727 ±159 ms. A custom software was developed in-house and used for visual inspection of each single pulse and manual correction of its calculated onset, if needed. The average value of the annotated onsets was 108 ±34 ms.

### 2.2. Pulse Onset Detection Methods

Over the last decade, various pulse onset detection methods have been proposed for the pulsatile signals other than CBFV, for example, ABP and PPG signals [26–31]. PPG signal (similar to CBFV) is inherently contaminated with various sources of noise and artifacts due to its measurement modality. Hence, in this study, we adapt three PPG pulse onset detection methods to obtain the onsets of the CBFV signal.

#### 2.2.1. Chen Method

**Figure 1:**
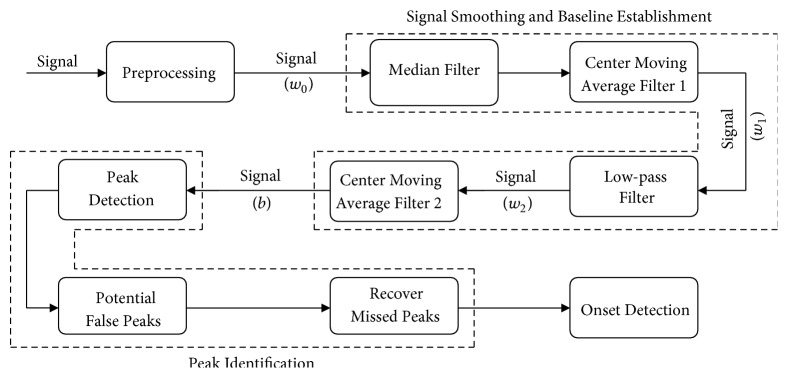
A block diagram of the major steps of* Chen method*.

In [27], Chen et al. have outlined a pulse onset detection method consisting of four major steps as depicted in Figure 1:

*Preprocessing.* In this step, the outlier data points with amplitudes more than *o*_*c*_ times median of the signal lasting for less than *i*_*c*_ seconds are identified and linearly interpolated to obtain signal *w*_0_. 

*Signal Smoothing and Baseline Establishment.* Power spectrum analysis is conducted on the detrended signal to estimate the heart rate frequency (*f*_*HR*_). For this purpose, the maximum power spectrum of the signal over the range of 0.8-3.0 Hz (corresponding to normal heart rates from 50 to 180 beats/min) is obtained. Then a cascade of four filtering techniques are employed to make the signal with less noise and sharp spikes (*w*_1_), fewer ectopic beats (*w*_2_), and an established baseline (*b*). The first two filters are a median filter and a center moving-average filter, both with the window sizes of 0.2/*f*_*HR*_, to remove noise and sharp spikes with frequencies above 5*f*_*HR*_. The third filter is a 3rd order low pass Butterworth filter with cutoff frequency of 1.5*f*_*HR*_ to remove the ectopic beats. Finally, a center moving-average filter with the window size 1.5/*f*_*HR*_ is applied to *w*_2_ to estimate a baseline *b* of the signal waveform. 

*Peak Identification.* The peak identification consists of three steps:

- *Peak detection*: First, the peaks of signal *w*_0_ are detected over the regions that auxiliary waveforms *w*_2_ are above *b*.
- *Identification of potential false peaks*: An adaptive thresholding on the amplitude of the peaks and the inter-beat time interval is employed to classify the detected peaks into potential false peaks or true peaks. A peak is labeled as potential false if its amplitude is less than half of certain percentile (*p*_*C*_) of the amplitude of all the peaks. Assuming that **t** is the vector of peak-to-peak time interval and median absolute deviation of the peak-to-peak interval is *MAD* = *median*(|**t** − *median*(**t**)|), peaks with intervals deviating from the median interval by more than *d*_*C*_ times *MAD* were also labeled as potential false peaks.
- Recover missed peaks: All the intervals between true peaks that include the potential false peaks are identified and carefully reexamined through an iterative procedure detailed in [27], in order to relocate the potential false peaks and/or recover missing peaks.

**Figure 2:**
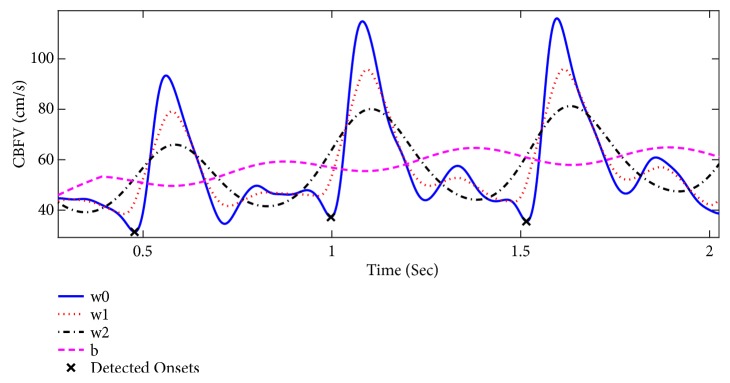
An example of pulse onset detection using* Chen method*.

*Onset Detection.* The onset of each pulse is detected by analyzing the signal between the peak of the corresponding pulse and its preceding one. For this purpose, the regions where the signal *w*_0_ is below both auxiliary waveforms *w*_2_ and *b* are identified (to ensure the independency of the detected onsets to the selection of the baseline). In case of having multiple ranges, they are ranked based on their lengths. Then the rightmost range from the top two ranges (the one closer to the peak) is selected. Finally, the onset is detected as the minimum of signal *w*_0_ over the selected range.  Figure 2 illustrates an example of pulse onset detection using* Chen method*. In [27], the default values for the parameters of the method were empirically set as *o*_*c*_ = 20, *i*_*c*_ = 0.2, *p*_*c*_ = 2/3, and *d*_*c*_ = 2.

#### 2.2.2. Asgari Method

**Figure 3:**
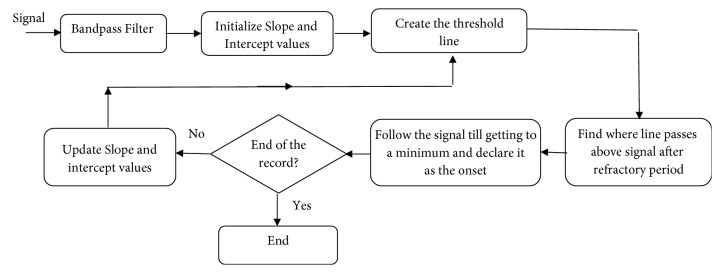
A block diagram of the major steps of* Asgari method*.

**Figure 4:**
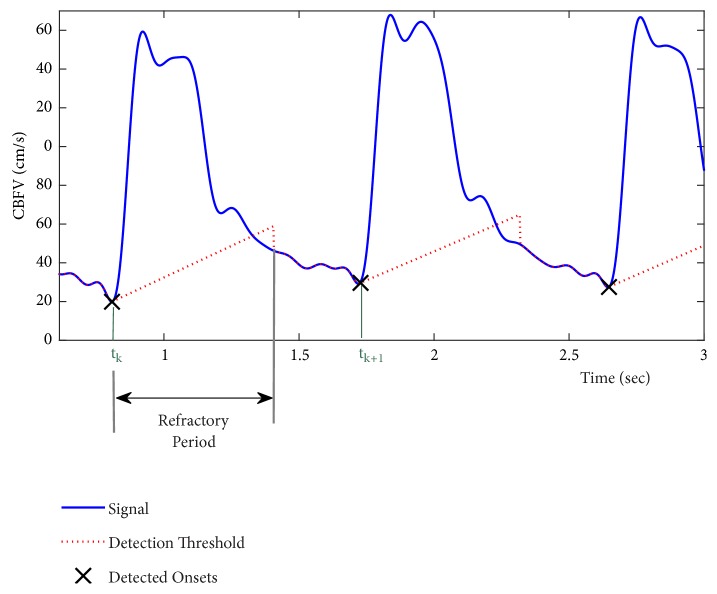
An example of pulse onset detection using* Asgari method*.

In [32], we proposed a modified version of a pulse onset detection method (*Shin method* [33] originally developed for PPG signals) to adapt it for CBFV pulses. The method is based on an iterative algorithm that calculates the location of the proceeding pulse onset (*t*_*k*+1_) based on that of the current pulse onset (*t*_*k*_) using an adaptive detection thresholding.  Figure 3 shows a flowchart of this algorithm. First a band-pass filter (0.5-10 Hz) is applied to the signal to remove the noise and artifacts. Then a threshold line is defined at *t*_*k*_ with the slope of

(1) $$\begin{array}{c} slope=\frac{\left(\left|V_{k}\right|+\sigma \right)r_{A}}{f}, \end{array}$$

where *V*_*k*_ is the amplitude of the current onset (*k* = 1,2, 3,…), *σ* is the standard deviation of the signal, *r*_*A*_ is a constant slope rate, and *f* is the sampling frequency. As Figure 4 shows, this threshold line continues for at least a refractory period of *τ* (to ensure that the detected onsets are at least *τ* seconds apart). As soon as the line passes above the signal amplitude, the threshold starts to follow the signal amplitude till a certain multiple number (*m*_*A*_) of average inter-beat time interval has been passed from the time of *t*_*k*_. Note that average inter-beat time interval (*η*) can be obtained as the reciprocal of estimated heart rate frequency at which the power spectrum of the signal is maximized (over 0.8-3Hz). Then the algorithm searches for the local minimums of the signal over the aforementioned searching range. In the case of having one minimum, that point is declared as the location of the next pulse onset *t*_*k*+1_. Otherwise, a center moving-average filter with the window size of *α*_*A*_ times *τ* is applied to smooth out the signal over the searching range. Then location of the first local minimum on the smoothed signal is identified. We hypothesize that this point is a good estimate for the location of the true onset. Therefore, starting from this location, we search for the minimum of the original (band-pass filtered) signal within a window size *τ* and declare that minimum point as the next pulse onset location *t*_*k*+1_.

For the next iteration, a new threshold line with an updated value of slope per (1) is defined at the location of the newly detected onset (*V*_*k*+1_). The above procedure is then repeated till the onsets of all the pulses (in the record) are identified. Note that the refractory period *τ* is defined as certain fraction (*t*_*A*_) of average inter-beat time interval *η*.

For the initialization step, the amplitude of the minimum of the signal over the first second of the data (let us call this amplitude *V*_0_) is obtained. Then the threshold line is defined with the slope and intercept values of (|*V*_0_ | + *σ*)*r*_*a*_/*f*and 0.2*V*_0_, respectively. In [32], default values for the parameters of the method were empirically set as *r*_*A*_ = 0.6, *m*_*A*_ = 2, *α*_*A*_ = 0.5, and *t*_*A*_ = 0.6.

#### 2.2.3. Farooq Method

**Figure 5:**
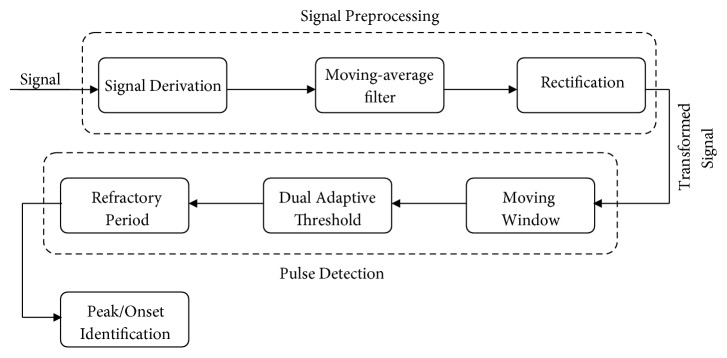
A block diagram of the major steps of* Farooq method*.

**Figure 6:**
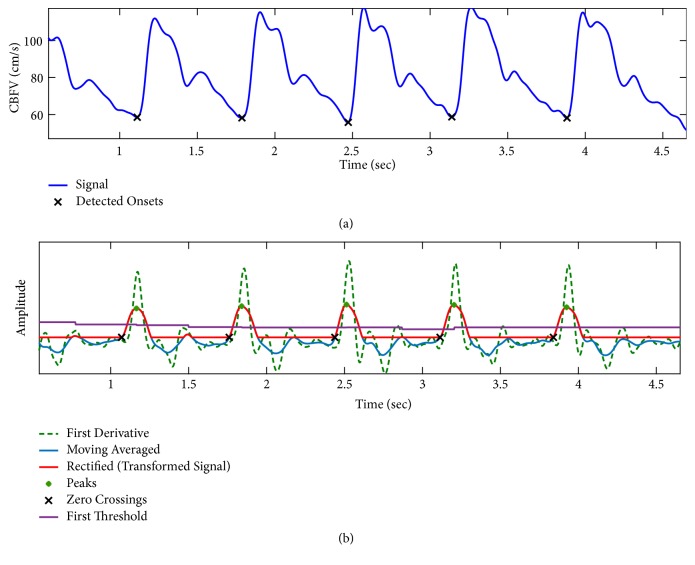
An example of pulse onset detection using* Farooq method*. (a) signal and detected onsets. (b) The auxiliary signals (derivative, moving-averaged, rectified) used to find the zero-crossings corresponding to pulse onsets.

In [29], Farooq et al. have outlined a pulse onset detection method which consists of three major steps as depicted in Figure 5: signal preprocessing, peak detection of the transformed signal, and onset detection of the original signal. For the preprocessing, first the derivative of the signal is calculated. Then a moving-average filter with time length of *l*_*F*_ seconds is applied. Finally, a rectifier is employed to zero the negative values of the signal. The output of the preprocessing step is a transformed signal with well-defined pulse separation, prominent peaks, and a reduced baseline wander (Figure 6).

At the next step, the peaks of the transformed signal are determined as follows: A moving window is applied and the maximum value of the transformed signal over each window is obtained. If the maximum value of any window is less than half that of the previous window, then the maximum of the previous window is declared as a peak. Following the determination of the peaks, the spurious peaks are identified and excluded from further processing by applying a dual adaptive thresholding on their amplitudes. Peaks with amplitudes less than a percentage (*p*_*F*_) of a threshold base value are excluded. Threshold base value is first initialized as the average of the amplitude of the peaks over the first ten seconds of data, and then it is made adaptive as the running average of the amplitude of the previous eight peaks. If this threshold fails to detect any peak, algorithm then searches back in time using a lower threshold by decreasing *p*_*F*_.

A refractory period of *δ*_*F*_ seconds is applied by excluding peaks that are less than *δ*_*F*_ seconds away from their preceding peak. Following the detection of the peaks on the transformed signal, the pulse onsets are identified by searching the transformed signal backwards starting from each detected valid peak till one gets to the first zero-crossing. The onset locations are then obtained by compensating for the delays that are introduced in the preprocessing step. In [29], default values for the parameters of the method were empirically set as *l*_*F*_ = 0.128, *p*_*Fa*_ = 0.5, *p*_*Fb*_ = 0.3, and *δ*_*F*_ = 0.25.

### 2.3. Data Analysis and Validation Protocol

All three methods (*Chen method*,* Asgari method*,* Farooq method*) were implemented in Matlab 2017b and applied to the CBFV data using the default values of the parameters of the corresponding methods as indicated in Section 2.2. The results of the onset detection were compared to those of the reference annotation and the following two performance benchmarks were calculated for each method: true positive rate (TPR) and positive productivity (PPV):

(2) TPR=TPTP+FN,PPV=TPTP+FP,

where true positive (TP) is a case when the detected onset is within a threshold value of *T* ms of the reference annotation, and a false positive (FP) case is when the algorithm falsely detects an onset in a location where there is no onset annotation.

To conduct a computational complexity analysis of the methods, the average running times of each method per pulse for all the CBFV records were calculated and compared using an Intel ® core ™ CPU@ 3.5 GHz, 32 GB RAM, 64-bit OS.

The sensitivity of the performance of each method to the selection of the parameter values was analyzed by calculating TPR and PPV when the value of each parameter was changed over a specific range while keeping the remaining parameters at their default values.  Table 1 summarizes the definition of each parameter, its default value, and its selected range of change for the sensitivity analysis of each method.

## 3. Results

**Figure 7:**
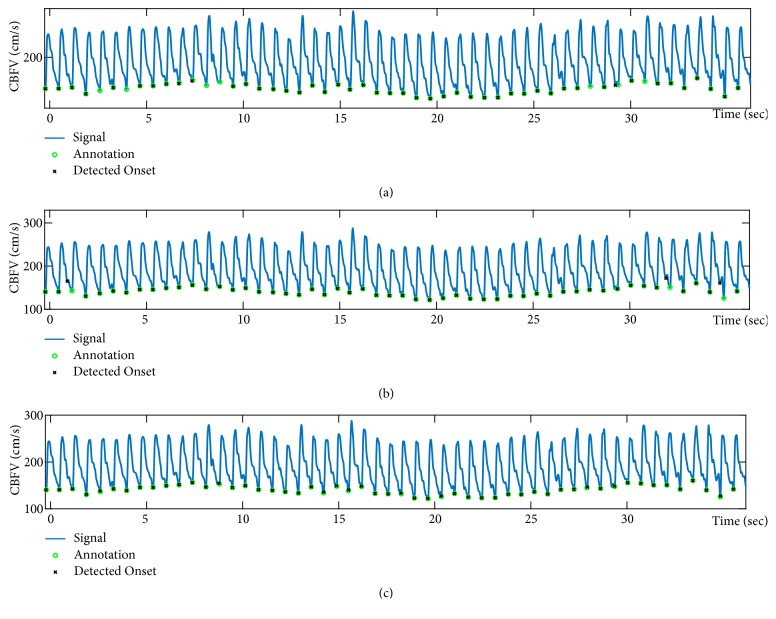
A sample case of CBFV onset detection results for the three methods: (a)* Chen method*; (b)* Asgari method*; (c)* Farooq method.*

Figure 7 shows a sample case of CBFV onset detection results for the three methods. While* Chen method* misses the identification of some of the onsets,* Asgari method* misallocates few other onsets. On the other hand,* Farooq method* is able to detect all of the onsets correctly.

Table 2 presents the results of CBFV onset detection in terms of TPR and PPV for the methods using the default values of their parameters for threshold values of *T* = 30, *T* = 20, and *T* = 10ms. We observe that for *T* = 30,* Farooq method* achieves the highest accuracy of detection with TPR=98.08% and PPV=97.75, while* Chen method* demonstrates the lowest accuracy with TPR=87.24% and PPV=95.91%. Similarly, for *T* = 20,* Farooq method* demonstrates a superior performance relative to the other methods with both TPR and PPV of above 95%. However, the detection accuracy of this method substantially declines to TPR=65.36% and PPV=65.14% when *T* = 10ms. In comparison to* Farooq method*, both* Asgari method* and* Chen method* performances remain less variant to the threshold value of *T*. Note that although the performance of* Chen method* has the lowest variability to the threshold value, it has the lowest TPR value relative to the other methods. Nevertheless,* Asgari method* shows both a reasonable level of accuracy in onset detection and a relatively low variability of the performance to the threshold value. In fact, for *T* = 30,* Asgari method* achieves TPR= 93.15% and PPV=93.30%, and it outperforms both* Farooq method* and* Chen method* with TPR=90.32% and PPV=90.46% when *T* = 10 ms.

Table 3 summarizes the results of the running time analysis for the three methods. We observe that while* Chen method* has the shortest running time of 1.1 ± 0.4 ms per pulse,* Asgari method's* running time is the longest (with a factor of 14) at 14.3 ± 3.9 ms per pulse. Farooq method also has a reasonable running time of 3.9 ± 1.5 ms per pulse.

**Figure 8:**
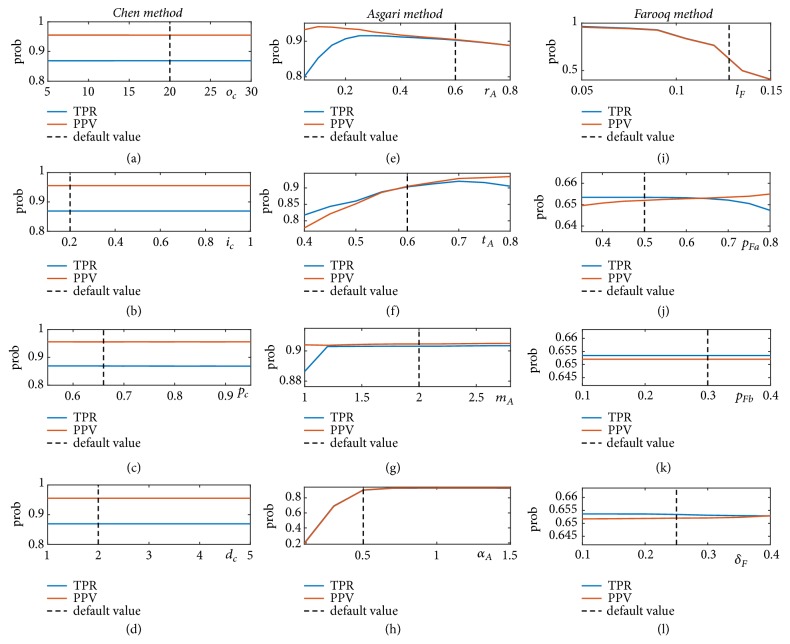
Sensitivity analysis of the methods to the selection of parameters values: (a)* Chen method o*_*c*_; (b)* Chen method i*_*c*_; (c)* Chen method p*_*c*_; (d)* Chen method d*_*c*_; (e)* Asgari method r*_*A*_; (f)* Asgari method t*_*A*_; (g)* Asgari method m*_*A*_; (h)* Asgari method α*_*A*_; (i)* Farooq method l*_*F*_; (g)* Farooq method p*_*Fa*_; (k)* Farooq method p*_*Fb*_; (l)* Farooq method δ*_*F*_.

Figure 8 presents the results of sensitivity analysis of all three methods to the selection of their parameters values (*T* = 10ms). We observe that the performance of* Chen method* has the least sensitivity to its parameters. For* Asgari method*, the accuracy of pulse onset detection relatively stays the same for the majority values of *m*_*A*_ and *α*_*A*_ per Figures 8(g) and 8(h). In fact, when *m*_*A*_ > 1.2 or *α*_*A*_ > 0.7, the onset detection achieves TPR and PPV of above 90%. As Figure 8(f) shows when *t*_*A*_ value increases, the performance of the onset detection improves, however when *t*_*A*_ > 0.7, TPR starts to decrease slightly. As a result,* Asgari method*'s performance will be optimized to above 90% for 0.6 ≤ *t*_*A*_ ≤ 0.7. From Figure 8(e) we observe that an increase in the value of slope rate *r*_*A*_ improves the TPR value at first, then TPR reaches a relative plateau of above 90% for 0.2 ≤ *r*_*A*_ ≤ 0.6. But then for larger values of slope rate, both TPR and PPV start to decline.

**Figure 9:**
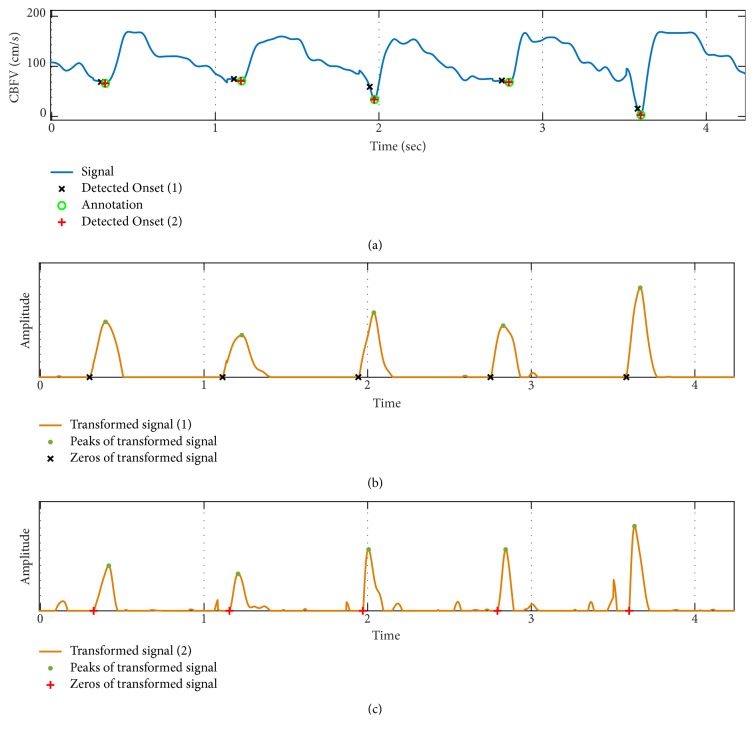
An example of CBFV onset detection results using Farooq method for two different moving-average window lengths (*l*_*F*_ = 0.128 for case 1, *l*_*F*_ = 0.06 for case 2): (a) Original signal; (b) transformed signal of case 1; (c) transformed signal of case 2.

As Figures 8(k) and 8(l) demonstrate, the accuracy of onset detection for* Farooq method* has low sensitivity to the choice of *p*_*Fb*_ and *δ*_*F*_ parameter values. However, per Figure 8(j), TPR slightly decreases for *p*_*Fa*_ ≥ 0.7. On the other hand, the length of the moving-average filter—applied to the derivative of the signal—can have a considerable effect on the performance of the onset detection. This is such that although for moving window length of less than 90ms both TPR and PPV stay above 92%, and when moving window length increases above 100 ms, the accuracy of onset detection declines considerably. In fact, as we observed from Table 2 before, for the default parameter value of *l*_*F*_ = 0.128, TPR and PPV of* Farooq method* can be as low as 65%.  Figure 9 shows the results of onset detection for a sample CBFV signal using* Farooq method* for window length of 128 ms (Case 1) versus 60 ms (Case 2). From Figures 9(b) and 9(c), we observe that for larger value of *l*_*F*_, width of the peaks on the transformed signal increases. Thus, the zero-crossings of the transformed signal occur further away from the true onset annotations resulting in higher level of error in localizations of the onsets.

Based on the results of sensitivity analysis, using the optimal values of the parameters for* Asgari method* (*r*_*A*_ = 0.3, *t*_*A*_ = 0.7, *m*_*A*_ = 2, *α*_*A*_ = 1) enhances the accuracy of pulse onset detection to TPR=91.65% and PPV= 93.62% for threshold *T* = 10ms. However, by using the optimal values of the parameters for* Farooq method* (*l*_*F*_ = 0.06, *p*_*Fa*_ = 0.6, *p*_*Fb*_ = 0.3, and *δ*_*F*_ = 0.25), the performance of onset detection increases substantially to TPR=97.06% and PPV=96.48%. These results indicate that by choosing appropriate parameter values,* Farooq method* outperforms the other two methods for the CBFV pulse onset detection.

## 4. Discussion

Low cost, simple, and noninvasiveness CBFV measurement has become a valuable clinical tool to study autoregulation with high temporal resolution. Furthermore, promising results for the noninvasive estimation of intracranial pressure have been recently obtained through the analysis of the CBFV (and ABP) and their relations to ICP pulse [14–20]. A reliable tracking of CBFV pulse morphology requires its accurate pulse delineation. Various methods have been proposed for the onset detection of the pulsatile signals other than CBFV. To facilitate the accurate delineation of CBFV pulse, the current work conducts a comprehensive comparative analysis of three popular onset detection methods for CBFV signal.

Among the three methods,* Chen method* showed the lowest accuracy in CBFV onset detection. The consistent low TPR value of* Chen method* (TPR~87% regardless of *T*) reflects the prevalence of missing onsets in this method. On the other hand,* Farooq method* showed the highest variability in performance with respect to the threshold *T*. In fact, while* Farooq method* achieved the highest performance for *T* = 30, its detection accuracy substantially downgraded when *T* = 10 ms. This observation shows that although* Farooq method* has the least number of missing onsets among the three methods, it results in the highest error value for estimation of pulse onset location (error is defined as temporal separation between annotated and detected onsets). In fact, an additional analysis revealed that average error values for* Chen method*,* Asgari method,* and* Farooq methods* are 3, 6, and 10 ms, respectively. Note that the average error of 10 ms for* Farooq method* is consistent with the observation of having a TPR and PPV of around 65% for *T* = 10ms (Table 2).

*Asgari method*'s average running time (~14 ms/pulse) was substantially higher than that of the other two methods. This is mainly due to the computationally expensive steps of the method (e.g., moving-average filtering to find the best minimum point on the searching range of each onset). Although all three methods showed a reasonable computational complexity,* Chen method* and* Farooq method* may be more appropriate choices for real-time applications in small scale, standalone, ubiquitous devices.

Our analysis of the methods' sensitivity to the selection of their parameter values showed that* Chen method* achieves the same level of performance regardless of the chosen values of its parameters. On the other hand, small values of slope rate in* Asgari method* make more of a flat threshold line which can result in missing some of the onsets. Similarly, a large value of the slope rate makes a steep threshold line that may cause the false identification of the dicrotic notch as the pulse onset [34]. Our results indicated that for 0.2 ≤ *r*_*A*_ ≤ 0.6,* Asgari method* achieves a TPR and PPV of above 90%. Since the refractory period in* Asgari method* is defined as a fraction of average inter-beat time interval (*t*_*A*_ × *η*), one can expect that (similar to *r*_*A*_) *t*_*A*_ should be also within a specific range for the method to perform well. In fact, our analysis revealed that* Asgari method*'s performance will be optimized to above 90% for 0.6 ≤ *t*_*A*_ ≤ 0.7.

For* Farooq method,* we observed that TPR of onset detection decreases slightly when the adaptive thresholding on the amplitude of the peaks are above 0.7. This could be justified by the fact that a higher threshold can result in missing detection of peaks with lower amplitudes. Our results also revealed that a window length of more than 100 ms for the moving-average filtering applied to the derivative of the signal considerably degrades the accuracy of onset detection. A long window (longer than the initial upslope of a typical pulse) can undermine enhancement of the upslope of the pulse. Furthermore, a longer window can increase width of the peaks on the transformed signal and as result, the detected onsets (zero-crossings) will be further away from the true onsets; i.e., the amount of error in onset detection increases. As we mentioned previously, the average error for the default value of *l*_*F*_ = 0.128 was 10 ms. Note that while this level of temporal separation between annotated and detected onsets does not significantly affect number of TP cases for threshold of *T* = 30 and *T* = 20ms, at least half of the detected onsets will be disqualified to be counted as a TP case for *T* = 10ms. This observation is consistent with the results of Table 2 showing that while* Farooq method*'s onset detection achieves a high accuracy for threshold of 30 and 20, it has a substantial decrease in its accuracy (to almost 65%) when threshold is set to 10 ms.

From the results of our sensitivity analysis, we realized that by using more appropriate parameter values*, Farooq method* can outperform the other two methods even for threshold as small as 10 ms. The need for adaptation of new parameter values for* Farooq method* is not surprising because the default values of these parameters were originally set for PPG onset detection in [29]. As the morphologies and baseline fluctuations of PPG signal may not be the same as those of CBFV, adjustment of the default values of parameters for CBFV onset detection seems inevitable.

Several limitations should be considered when interpreting the results of this study. The dataset used for our analyses had a limited size and included CBFV signal of only subarachnoid hemorrhage patients. Employment of a larger dataset of CBFV signals from healthy controls as well as different pathophysiology could enhance the reliability of the results. Another limitation was the inherent challenge of analyzing and identifying the ground truth for the onset of noisy CBFV pulses. Although we used the ECG R-peak locations to help with identification of foot (minimum point) of each pulse, the indicated ground truth for some of the noisy pulses might not be sufficiently reliable. These limitations could have biased our results.

## 5. Conclusion

We conducted a comprehensive analysis of three state-of-the-art pulse onset detection methods for CBFV signal. Our analysis revealed the need for further adjustment of the parameters of the methods to enhance the accuracy of CBFV pulse onset detection. The results showed that the best method (*Farooq method*) can achieve a TPR and PPV of above 96% for small error threshold value of 10 ms. This method has a reasonable running time of less than 4 ms/pulse. Thus, it can be used reliably for the CBFV onset detection.

## Figures and Tables

**Table 1:** Definition of each parameter, its default value, and its selected range of change ([minimum value, maximum value]) that were employed for the sensitivity analysis of each method.

Method	Parameter (default value)	Definition	Range of Change
*Chen Method*	*o*_*c*_ = 20	Signal outliers with amplitudes more than *o*_*c*_ times median of the signal lasting for less than *i*_*c*_ second are linearly interpolated.	[5 30]
	*i*_*c*_ = 0.2	Signal outliers with amplitudes more than *o*_*c*_ times median of the signal lasting for less than *i*_*c*_ second are linearly interpolated.	[0.1 1]
	*p*_*c*_ = 66%	Signal peak is labeled as potential false if its amplitude is less than half of (*p*_*C*_) percentile of the amplitude of all the peaks.	[55% 100%]
	*d*_*c*_ = 2	Signal peaks with intervals deviating from the median interval by more than *d*_*C*_ times *MAD* are labeled as potential false peaks	[1 5]

*Asgari Method*	*r*_*A*_ = 0.6	Constant slope rate in Eq. (1)	[0 0.8]
	*t*_*A*_ = 0.6	Refractory period *τ* is defined as certain fraction (*t*_*A*_) of average inter-beat time interval *η*.	[0.4 0.8]
	*m*_*A*_ = 2	When threshold line passes above the signal amplitude (after refractory period), the algorithm starts to search for the next pulse onset till an interval time equal to *m*_*A*_ × *η* (from the current onset) has been passed.	[1 3]
	*α*_*A*_ = 0.5	A moving-average filter with window size of *α*_*A*_ × *τ* is applied to smooth out the data over the searching range.	[0.1 1.5]

*Farooq Method*	*l*_*F*_ = 0.128	A moving-average filter with time length of *l*_*F*_ seconds is applied to the derivative of the signal.	[0.05 0.15]
	*p*_*Fa*_ = 0.5	Peaks with amplitudes less than a fraction (*p*_*Fa*_) of a threshold base value are excluded	[0.35 0.8]
	*p*_*Fb*_ = 0.3	If *p*_*Fa*_ fails to detect any peak, algorithm then searches back in time using *p*_*Fb*_.	[0.1 0.45]
	*δ*_*F*_ = 0.25	Peaks that are less than *δ*_*F*_ seconds away from their preceding peak are excluded from further processing.	[0.1 0.4]

**Table 2:** Comparison of the performances of different CBFV onset detection methods in terms of TPR (%) and PPV (%) using the default values of their parameters and for threshold of *T* = 30, *T* = 20 and *T* = 10ms.

	*T* = 30ms		*T* = 20ms		*T* = 10ms	
Method	TPR	PPV	TPR	PPV	TPR	PPV
*Chen method*	87.24	95.91	87.05	95.70	86.91	95.55
*Asgari method*	93.15	93.30	92.81	92.86	90.32	90.46
*Farooq method*	98.08	97.75	95.96	95.63	65.36	65.14

**Table 3:** Comparison of the average running times of different CBFV onset detection methods.

Method	*Chen Method*	*Asgari Method*	*Farooq Method*
Average running time (ms/ pulse)	1.1 ± 0.4	14.3 ± 3.9	3.9 ± 1.5
